# Semi-supervised segmentation of retinoblastoma tumors in fundus images

**DOI:** 10.1038/s41598-023-39909-6

**Published:** 2023-08-10

**Authors:** Amir Rahdar, Mohamad Javad Ahmadi, Masood Naseripour, Abtin Akhtari, Ahad Sedaghat, Vahid Zare Hosseinabadi, Parsa Yarmohamadi, Samin Hajihasani, Reza Mirshahi

**Affiliations:** 1 Chashmyar Company, Tehran, Iran; 2 Eye Research Center, The Five Senses Institute, Rassoul Akram Hospital, Iran University of Medical Sciences, Tehran, Iran; 3 School of Medicine, Shahid Beheshti University of Medical Sciences, Tehran, Iran; 4 Young Researchers and Elite Club, Tehran Medical Sciences, Islamic Azad University, Tehran, Iran; 5 Student Research Committee, Shahrood Branch, Islamic Azad University, Shahrood, Iran

**Keywords:** Cancer imaging, Computational science

## Abstract

Retinoblastoma is a rare form of cancer that predominantly affects young children as the primary intraocular malignancy. Studies conducted in developed and some developing countries have revealed that early detection can successfully cure over 90% of children with retinoblastoma. An unusual white reflection in the pupil is the most common presenting symptom. Depending on the tumor size, shape, and location, medical experts may opt for different approaches and treatments, with the results varying significantly due to the high reliance on prior knowledge and experience. This study aims to present a model based on semi-supervised machine learning that will yield segmentation results comparable to those achieved by medical experts. First, the Gaussian mixture model is utilized to detect abnormalities in approximately 4200 fundus images. Due to the high computational cost of this process, the results of this approach are then used to train a cost-effective model for the same purpose. The proposed model demonstrated promising results in extracting highly detailed boundaries in fundus images. Using the Sørensen–Dice coefficient as the comparison metric for segmentation tasks, an average accuracy of 93% on evaluation data was achieved.

## Introduction

Retinoblastoma is a rare type of cancer that primarily affects young children and accounts for 3% of childhood cancer cases globally, with a prevalence of 1 in 18,000 live births^1^. The disease is classified into five groups from A to E, with the prognosis worsening from group A to E. Retinoblastoma is caused by inactivating mutations of both alleles of the retinoblastoma (RB1) gene, located on chromosome 13q14, and occurs in two forms: germline mutations and non-heritable mutations^2–4^. Symptoms of retinoblastoma include poor vision in older children, strabismus, and white color in the pupil (leukocoria), which indicates the absence of a red reflex due to retinal involvement on the affected side^5,6^. Treatment options include enucleation, systemic chemotherapy, radiotherapy, and local therapies^7^, with the primary goal being to save the patient's life while preserving their eye and vision. Fundus photography is an essential ancillary test during diagnosis and follow-up, enabling practitioners to examine a patient's retina, detect abnormalities, assess retinal findings, and track changes in lesions following treatment^8^.

In the context of tumor segmentation, machine learning and image processing have been shown to yield excellent results for liver^9^ and lung^10^ cancers. For example, residual networks have proven to be effective in identifying cancerous pixels in X-Ray scans for liver cancer, while non-data-driven methods such as thresholding have produced outstanding results for lung tumor segmentation.

Supervised learning is a subfield of machine learning that relies on prior knowledge of both input and output data. A sufficient amount of labeled data is required to create or train a supervised model. Alternatively, unsupervised learning involves training machine learning models without any knowledge of labels. Then again, semi-supervised learning describes a situation where there is limited knowledge of labels for a small portion of the data but no recorded labels for the remaining data. Using machine learning for the segmentation of retinal lesions is a task that has been explored in various ophthalmology-related studies^11,12^.

In the context of tumor segmentation, various machine-learning methods are used to detect abnormal areas depending on the data. In recent years, most of these methods have been based on deep learning. This method has outperformed older approaches in pixel labeling for tumor segmentation, resulting in more precise detection of tumor edges and boundaries^13^. However, this problem has only been considered in a limited number of studies due to the lack of sufficient labeled images (pixel-wise), leading some studies to rely mainly on image processing^5^ or use relatively small datasets^14^. Although image processing-based approaches for retinoblastoma detection and segmentation have achieved acceptable accuracy^15^, most of these methods lack proper generalization due to their reliance on the designer's limited expertise or the small sample size. In contrast, some advanced studies have utilized fairly large amounts of data for training machine learning-based models and achieved high recognition rates and segmentation accuracy; however, the accuracy of training data labels for each pixel can lead to smooth boundaries and edges in both training and predicted labels, resulting in inaccuracies^16,17^.

To address the aforementioned challenges, this study aims to utilize a reasonably large dataset for retinoblastoma tumor segmentation, in which none of the samples have been previously labeled. To label and process this data, an unsupervised method is used to label each pixel of each fundus image. Then, the results are manually refined based on the medical experts' prior knowledge, making the entire process semi-supervised. Finally, a model with two different types of output is proposed to eliminate the need for manual intervention and create a fully automated process.

## Method

The current study was approved by the Ethical Committee of the Iran University of Medical Sciences (IR.IUMS.FMD.REC.1398.292) and was conducted in accordance with the principles outlined in the Declaration of Helsinki. Additionally, the parents or legal guardians of all patients provided informed consent prior to participation in the study.

### Labeling pixels using unsupervised learning

In this section, we will explain the process of labeling pixels of fundus images obtained by the RetCam wide-field digital imaging system (Clarity Medical Systems, Pleasanton, California, USA) for detecting retinoblastoma. Due to the various manifestations of tumors in fundus images, healthy pixels were first detected, and then the undetected regions were considered as pixels associated with a tumor. This process was accomplished by targeting color profiles or the density of affected areas. Meanwhile, healthy cases share a more similar visualization in fundus images, making them more appropriate to consider as a ground truth. To satisfy the conditions above in all targeted images, it is necessary to ensure that the majority of the areas are healthy to demonstrate the general features of healthy texture in fundus images. To achieve this, the process concatenates the targeted image with three selected healthy fundus images on the horizontal (or vertical) dimension. This step ensures that healthy areas are the majority of data and that these areas properly visualize the healthy aspects of the retina in the fundus image. By doing so, assuming a size of 320 by 480 pixels for one image, an array with the shape of 1280 by 480 pixels was obtained.

In this study, the Gaussian Mixture Model (GMM) was chosen as the clustering algorithm over other well-known methods, such as K-means, for its capability of drawing elliptical boundaries, resulting in more precise clustering. Additionally, GMM is a probabilistic algorithm that assigns probabilities to each sample or point, expressing the strength of the model's belief in the assignment to a specific cluster, in contrast to K-means, which assigns clusters with certainty.

A Gaussian Mixture Model (GMM) is a function that consists of multiple Gaussians, each described by the parameter $k$, where $k$ belongs to the set $\{\mathrm{1,2},3,...,K\}$, and $K$ is the number of clusters in the selected dataset. Each Gaussian in the mixture is characterized by three parameters: $\{\mu ,\Sigma ,\pi \}$. The mean centroid of the cluster is represented by $\mu$, the covariance of the cluster by $\Sigma$, and the mixing probability of the cluster by $\pi$. In simpler terms, the GMM algorithm ensures that each Gaussian accurately fits the data points belonging to each cluster by calculating the optimal values of $\{\mu ,\Sigma ,\pi \}$. These values are determined by taking the derivative of (1) with respect to the means and variances, where $x$ represents a data point, and $D$ represents the number of dimensions in each data point.

1 $$-\frac{D}{2}\mathit{ln}2\pi -\frac{1}{2}\mathit{ln}\Sigma -\frac{1}{2}(x-\mu {)}^{T}{\Sigma }^{-1}(x-\mu )$$

By solving the optimization problem, GMM provides solutions in the form of Eqs. (2–4), where $N$ is the number of data points, ${z}_{nk}$ is the probability that a data point ${x}_{n}$ belongs to Gaussian $k$, and $\gamma ({z}_{nk})=p({z}_{k}=1|{x}_{n})$. Using these equations in iteration $k$, new values of $\{\mu ,\Sigma ,\pi \}$ are calculated based on (1). For the next iteration, a new value of $\gamma ({z}_{nk})$ is computed using the normal distribution function and Eq. (5).

2 $${\pi }_{k}=\frac{1}{N}{\sum }_{n=1}^{N}\gamma ({z}_{nk})$$

3 $${\mu }_{k}=[{\sum }_{n=1}^{N}{x}_{n}\gamma ({z}_{nk})]/[{\sum }_{n=1}^{N}\gamma ({z}_{nk})]$$

4 $${\Sigma }_{k}=[{\sum }_{n=1}^{N}({x}_{n}-{\mu }_{k})({x}_{n}-{\mu }_{k}{)}^{T}\gamma ({z}_{nk})]/[{\sum }_{n=1}^{N}\gamma ({z}_{nk})]$$

5 $$\gamma ({z}_{nk})={\pi }_{k}{\rm N}({x}_{n}|{\mu }_{k},{\Sigma }_{k})/({\sum }_{k=1}^{K}{\pi }_{k}{\rm N}({x}_{n}|{\mu }_{k},{\Sigma }_{k}))$$

**Figure 1:**
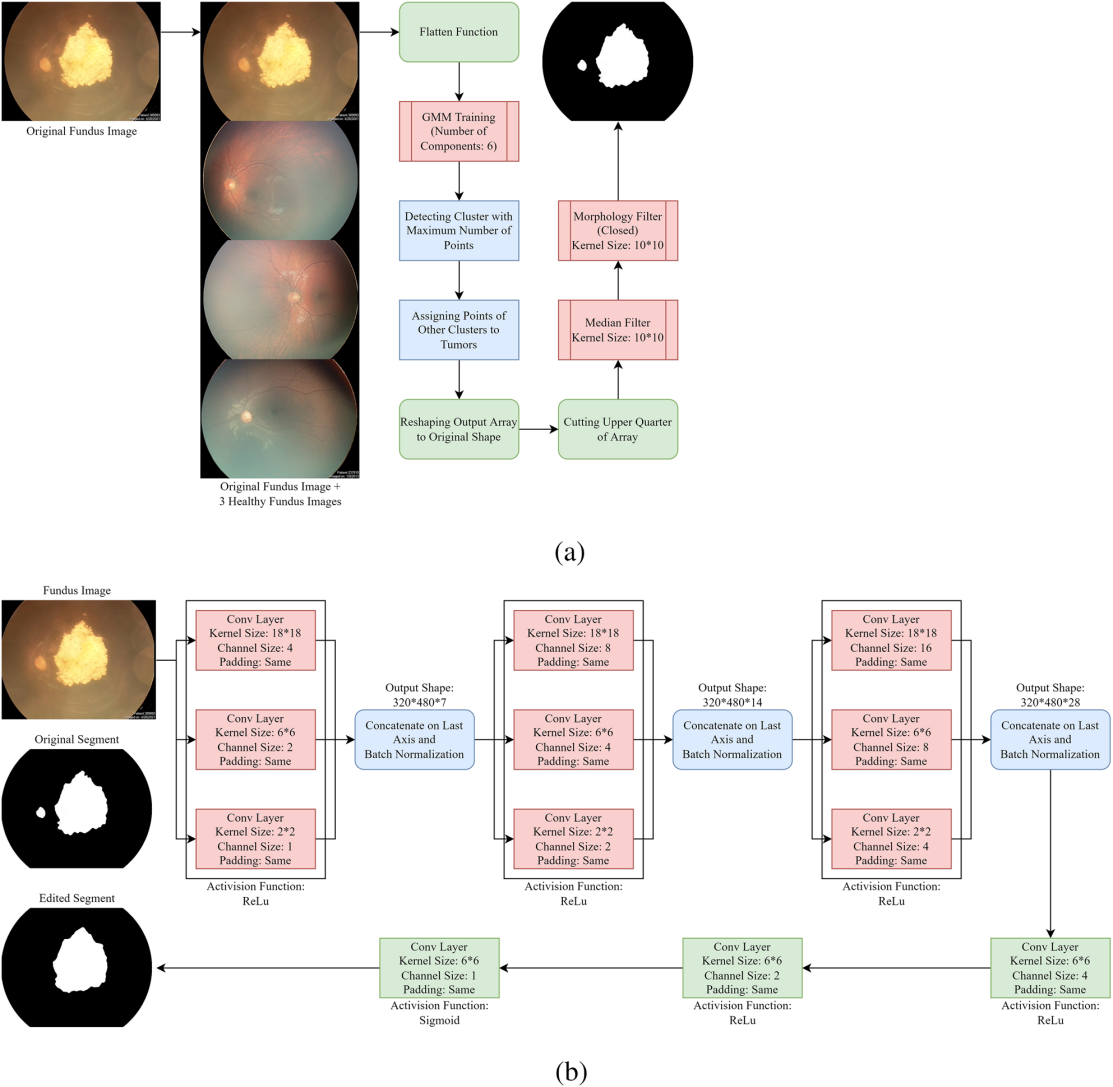
(**a**) The complete clustering process (first approach) of segmenting tumors. During this process, the GMM algorithm groups all values within the flattened input array into one of six clusters. The cluster with the highest number of members is then designated as representing healthy pixels, while the other clusters are categorized as suspected pixels. Next, the resulting array is reshaped into a 2D array, and the upper half of this new representation is subjected to post-processing using median and morphology filters. (**b**) Suggested convolutional neural network for refinement of segmentation results. The proposed neural network architecture comprises three individual subnetworks, each consisting of three parallel convolutional layers. The output of each layer is then combined to form a unified array, which serves as the input for the subsequent subnetwork. This hierarchical structure allows the network to extract increasingly complex features and patterns from the input data, enabling it to learn high-level representations of the segmented images. Moreover, the parallel architecture of the subnetworks helps speed up the computation process while maintaining the accuracy of the predictions.

At the end of the clustering process, the cluster with the largest number of points is designated as healthy, while the remaining clusters are merged into a single, larger cluster representing unhealthy areas. Figure 1a provides a visual representation of the entire process. Using this approach, clusters that do not correspond to healthy areas are consolidated into a larger area, and small regions of that area are removed using morphology filtering in close form. A median filter is utilized to accomplish this step.

### The refinement of segmentation process

This study proposed a supervised process using deep neural networks to address the issue of the GMM algorithm recognizing the optic disc as an anomaly and to refine the segmentation process for all fundus images. The clustering method in the previous step was performed individually on each fundus image, and the model did not learn from other photos. The refinement process involved manually removing signs of the optic disc in each segmentation result based on general knowledge of its shape. This step was only necessary for a small number of cases, as the optic disc did not exist in most images or was already ignored by the model. To accomplish this, a multi-layer convolutional neural network was suggested due to its ability to process multi-dimensional arrays. The network consists of three subnetworks, each with three parallel convolutional layers. The output of each layer was merged into one array and used as input for the next sub-network (Fig. 1b).

## Results

### Data and simulation details

In order to evaluate the effectiveness of the proposed approach, a total of 4176 fundus images of both healthy patients and patients with retinoblastoma were utilized for tumor segmentation. All images were resized to a dimension of 320 × 480, and a grayscale version was employed to train the GMM. The experiment was conducted on a laptop equipped with an 11th Gen Intel(R) Core (TM) i7-11370H CPU, 40 GB of physical DDR4 RAM, and an Nvidia RTX 3070 GPU with 8GB VRAM. The ophthalmologists who participated in this research also provided their expertise in areas such as filtering out weak results obtained from the GMM-based method, as well as generating ground truth data for tumor segmentation in the evaluation phase.

### Visual results

**Figure 2:**
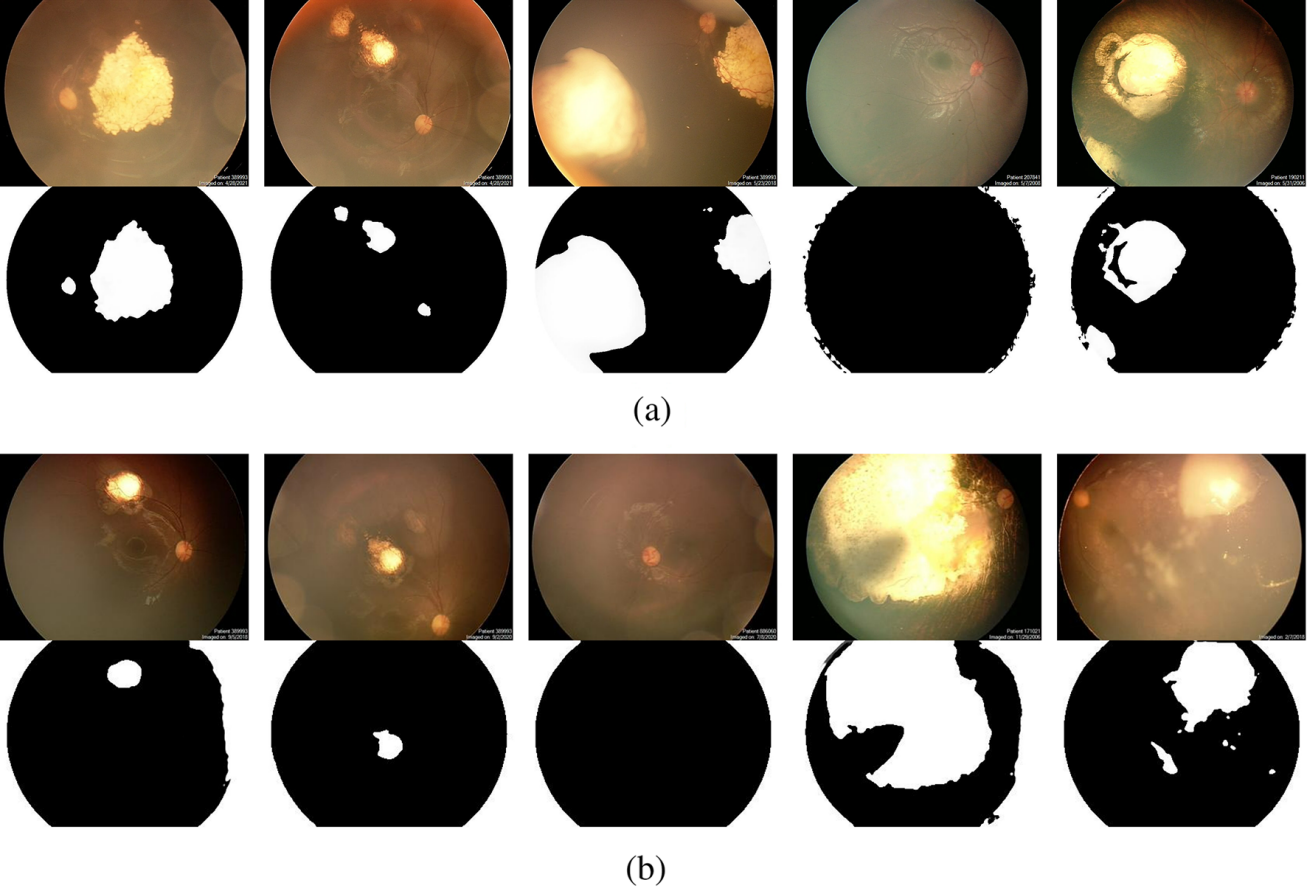
(**a**) Sample of results regarding segmentation of tumors, using clustering. Given the nature of this method, the achieved boundaries are expected to be as precise as possible. Moreover, using more powerful hardware capable of processing fundus images with higher resolution, smaller signs of retinoblastoma can be detected. However, when compared to the suggested supervised method, the computational cost of this approach is significantly higher. (**b**) Successful Results of segmentation refinement based on the first approach, where the model is trained using original images as input data and manually edited outputs of unsupervised method as output data. Note that value of each pixel in final results is mapped to one or zero, using a threshold of 0.5. The suggested method has successfully achieved its primary objective for the majority of candidate images. However, it should be noted that this method is not foolproof, which may be due to the small size of the network or the low resolution of input images. Further research and development may be required to overcome these limitations and improve the reliability of the method.

As previously mentioned, GMM was utilized as the primary clustering method for creating the original ground truth for tumor segmentation. In this study, the number of clusters (or components) was set to six, and the number of initializations was set to twenty-five. These values were selected and optimized through trial and error, and minor adjustments to these values did not significantly alter the final clustering results. Of all the results, 3279 outputs were considered to be properly segmented based on a medical expert's evaluation. However, a significant number of cases exhibited the optic disc in fundus images being erroneously segmented as a tumor or, at least, a portion of it being segmented (as depicted in Fig. 2a). Another drawback of this approach was the considerable computational cost of the process, requiring a significant amount of time to provide an output. To be more precise, clustering pixels of each image using said hardware takes nearly 2 min. As previously mentioned, deep neural networks were specifically chosen to address this issue.

Two different approaches were selected to train the network illustrated in Fig. 1b. Both approaches used RMSProp as the learning algorithm, with a learning rate of 0.001. Ten percent of the data (3279 samples) was randomly selected for evaluating the trained model. From the remaining 90% of the data, 10% was selected for validation, and 90% (81% of the total data) was used to train the model. In both scenarios, the training process was performed using one sample per training step, i.e., the batch size was set to one. This choice was also made based on trial and error and studying the final results. However, since each fundus image exhibits unique features, using the average loss of multiple images for updating trainable parameters is not recommended.

As previously mentioned, two approaches were selected for training the designed network, each prioritizing a different aspect. One approach focused on achieving highly detailed boundaries for tumors, while the other aimed to present a measure of each pixel’s importance in the input image. Since the output of the clustering approach is a binary image, it is possible to force the neural network to create only zeros or ones for each pixel by using mean absolute error as a loss function. By the end of the training process, the threshold for the output array was set to 0.5, where values above the threshold were mapped to one, and values under the threshold were mapped to zero. If the training process reaches an acceptable loss value, the process also reaches the most accurate boundaries for tumors. Some results achieved by this method are illustrated in Fig. 2b. Finally, studying all evaluation results showed that, despite not achieving perfect results, the achieved boundaries and conditions for removing the optic disc from the segmentation results reached a promising level, exceeding expectations compared to prior studies.

**Figure 3:**
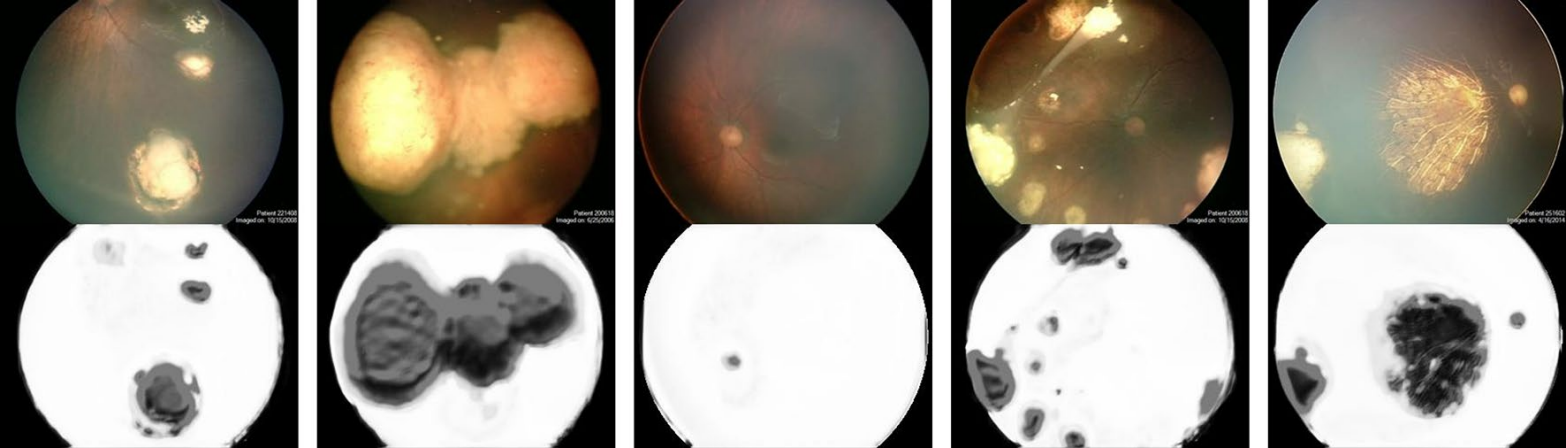
Results of segmentation refinement, based on the second approach, where the model is trained using original images as input data and manually edited outputs of unsupervised method as output data, while no thresholding step is performed. This approach is equally fast compared to its predecessor, but it can provide more detailed information about the height of detected tumors. The method can be further improved by increasing the resolution of input images and enhancing computational speed, resulting in the most accurate and detailed segmentation of retinoblastoma in this study. Nevertheless, this method may face challenges in detecting the optic disc as an anomaly compared to the introduced supervised approach.

The second approach aims to present an importance-based segmentation. Initial simulations revealed that despite using binary images for the network's output in the training process, the trained network aimed to create outputs with different contrast values by using binary cross-entropy as a loss function. The final results indicated that the segmented discs had a lighter gray tone than the darker tone of tumor segments, despite occasionally showing optic discs. It is noteworthy that no information regarding the importance of each pixel was provided during the training process, given that the outputs were binary. The model demonstrated an impressive level of understanding regarding its inputs. Figure 3 illustrates some results regarding this approach.

**Figure 4:**
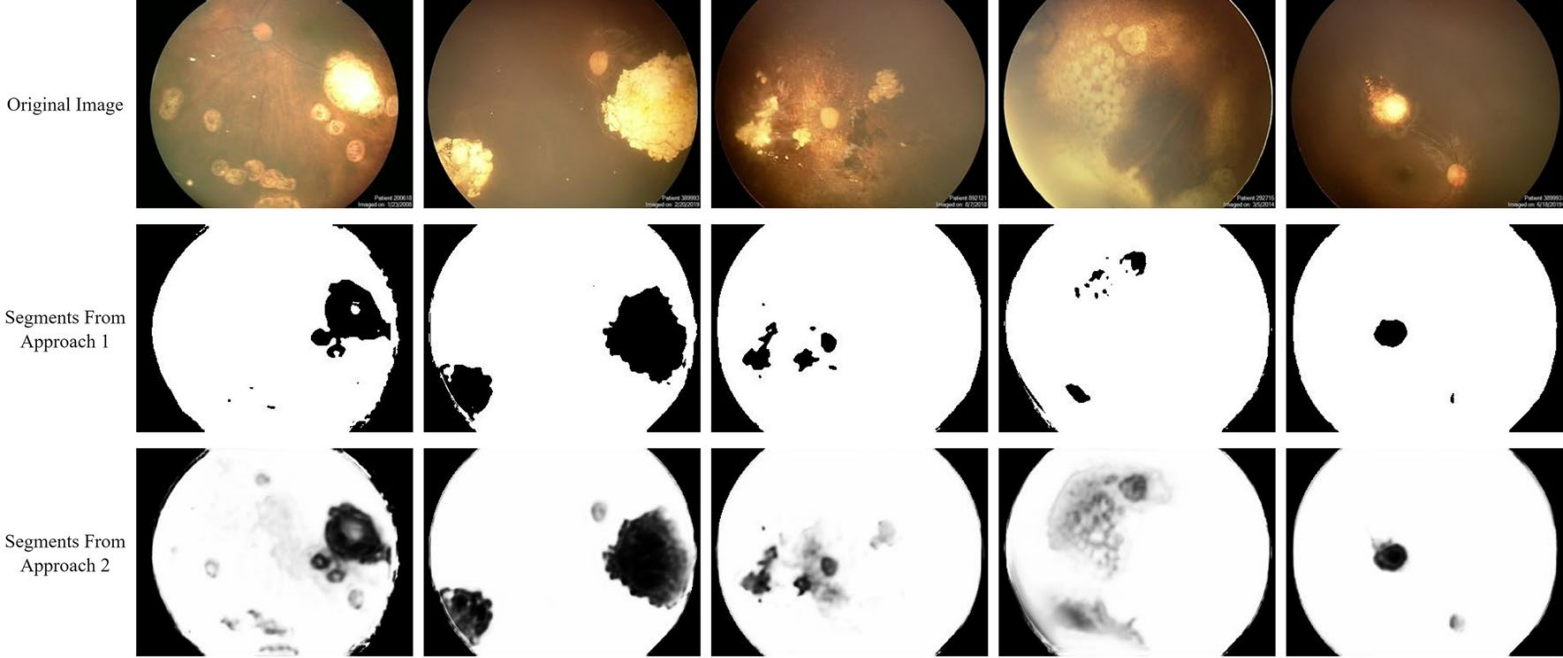
Comparison of results regarding two introduced approaches, where first method includes a thresholding step for each pixel in output image and second method presents the exact value of each pixel, which is a value between zero and one. Note that both methods share the same structure. Although the majority of the results obtained from the proposed method were satisfactory, there were some outputs with inaccurate tumor detection, as shown in the figure. Interestingly, the use of non-binary output for each pixel improved the tumor detection, suggesting the potential for further refinement through a proper filtering process based on the value of each pixel. With such improvements, the quality of the final output can be further enhanced.

**Figure 5:**
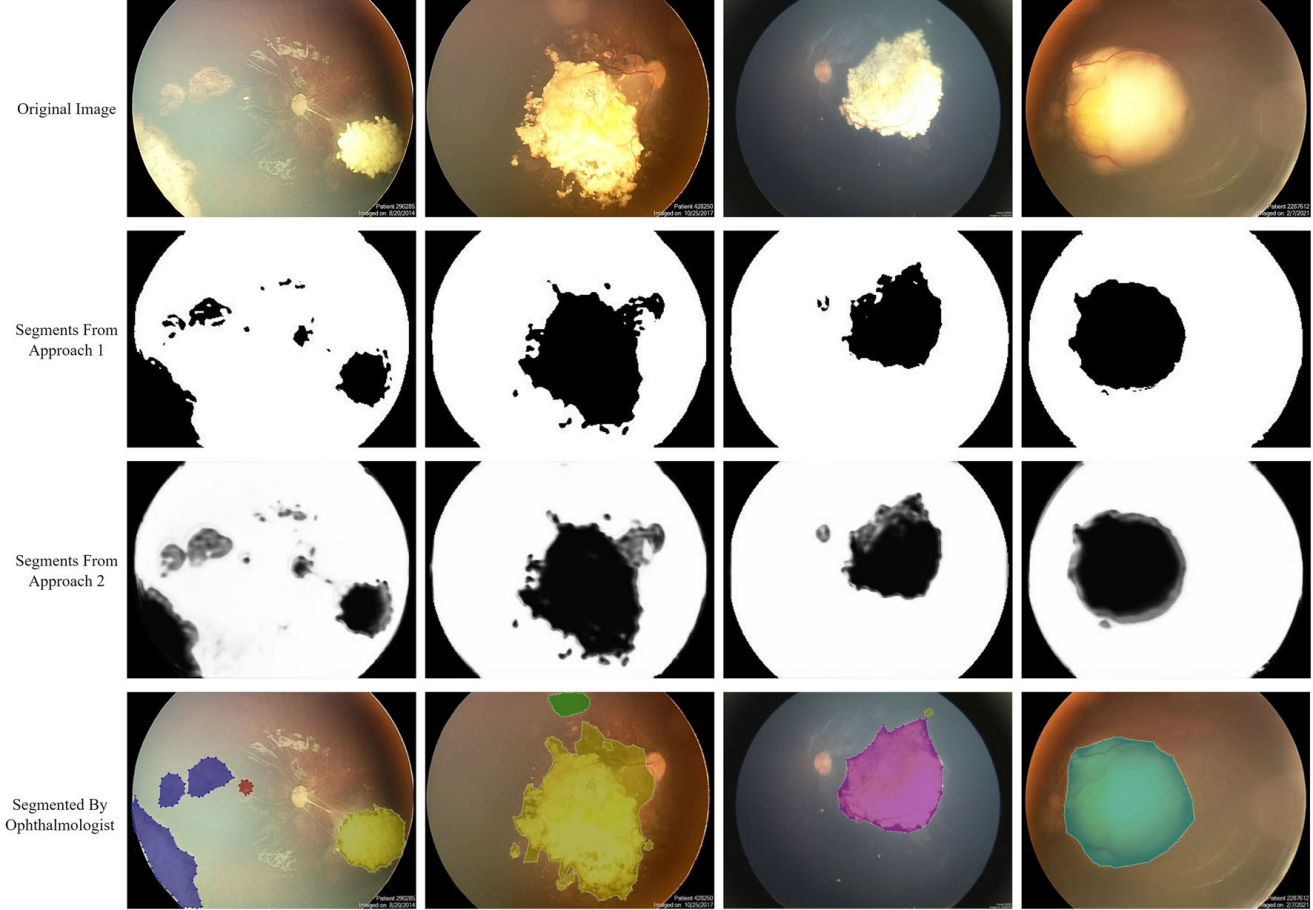
Results of suggested approaches (upper images) compared to segments identified by ophthalmologists (lower images). Extra details unrelated to tumors have been highlighted in the first case (from the left) for practical application. Results of the second case from the left indicate that while the tumor has been detected in the upper image, its shade is quite light and needs to be enhanced.

As previously mentioned, two approaches were selected for training the designed network. Each approach was chosen based on a specific priority, one focusing on achieving highly detailed tumor boundaries and the other on presenting a measure of pixel importance in the input image. By using mean absolute error as a loss function, the first approach was able to equate each pixel to zero or one, allowing for accurate tumor boundary detection. As Fig. 2b illustrates, the achieved boundaries and the successful removal of the optic disc from segmentation results exceeded expectations. The second approach used binary cross-entropy as a loss function to create outputs with different contrast values. Even though the segmented discs showed optic discs in some cases, they demonstrated a lighter grey tone compared to the darker tone of tumor segments. Despite not providing any information about the importance of each pixel during the training process, the model reached a level of deep understanding regarding its inputs, as illustrated in Fig. 3. After comparing the results of both approaches, it is evident that the first approach draws more absolute boundaries for tumors and is more successful in removing the optic disc. However, the second approach provides richer and more detailed results, including a measure of depth, despite its weaker performance in removing the optic disc. Ultimately, choosing one of these two approaches depends on individual preference, as both are capable of properly demonstrating retinoblastoma. Figure 4 illustrates a fair comparison of both approaches in several complex cases. Furthermore, the suggested methods' results were compared with the results of tumor segmentation performed by ophthalmologists using LabelMe software, as presented in Fig. 5. While the higher resolution of original images provides both machine learning-based models and ophthalmologists with more accurate information, the improvement in machine learning-based models is more noticeable. It is worth noting that the visual results presented in this research show outputs that are far from perfect. While the model provides more accurate segmentations for most inputs, it was intended to demonstrate the segmentation results for more challenging inputs to provide more realistic and fair results.

### Numerical results

We use the Sørensen–Dice coefficient (SDC), a widely used measure for comparing model-based segmentation results with ground truth in image processing, to provide a more effective comparison. This equation is shown in (6), where $X$ and $Y$ represent the segmentation result and the ground truth, respectively, and $TP$, $FP$, $FN$_,_ and $TN$ denote the numbers of true positives, false positives, false negatives, and true negatives, respectively. The Sørensen–Dice coefficient provides a normalized ratio of similarities between the two input arrays (images), taking into account their size. The resulting values range from zero to one, with higher values indicating better performance. Table 1 shows the values obtained for the cases presented in Fig. 5, using the second approach.

**Table 1:** Value of Sørensen–Dice coefficient for fundus images in Fig. 5, based on the results of second approach.

Image	SDC value
Image 1	0.67
Image 2	0.86
Image 3	0.84
Image 4	0.96

6 $$SDC=\frac{2\left|X\cap Y\right|}{\left|X\right|+\left|Y\right|}=\frac{2TP}{2TP+FP+FN}$$

7 $$Sensitivity=\frac{TP}{TP+FN}$$

8 $$Specificity=\frac{TN}{TN+FP}$$

9 $$Accuracy=\frac{TP+TN}{(TP+FN)+(TN+FP)}$$

To conduct this study, Table 2 presents the minimum, maximum, and average values of the SDC, based on the results of the second approach. Although the Sørensen–Dice coefficient is a comprehensive descriptor for image segmentation results, some studies preferred to use simpler metrics. For example, segmentation results can be compared with ground truth using the Sensitivity, Specificity, and Accuracy metrics^18^, described in (7–9), respectively. Table 3 shows the average value of each metric for the evaluation data, based on the second approach, indicating that the presented model performed acceptably. Please note that RetCam images were also used in the evaluation phase of the study, and that all results based on numerical metrics were obtained by comparing the output of our proposed model with the segmented areas extracted from the original RetCam images using LabelMe software, as verified by medical experts.

**Table 2:** Value of Sørensen–Dice coefficient for evaluation fundus images, based on the result of second approach.

Metric	SDC value
Least value	0.06
Highest value	0.98
Average value	0.93

**Table 3:** Average value of sensitivity, specificity and accuracy, based on the results of second approach.

Metric	Value
Sensitivity	0.92
Specificity	0.77
Accuracy	0.89

As mentioned earlier, the precision of tumor segmentation is directly related to the quality of the image. Therefore, as the size of the fundus image increases, the need for more powerful hardware becomes unavoidable. However, the proposed methods' results are easily compared with the work of ophthalmologists and medical experts and will be even more precise in detail if more powerful hardware is available. It is worth noting that despite the few differences between the results of the clustering process and the final results, clustering each image using the mentioned hardware took nearly 2 min, while the trained model processed 328 evaluation images and segmented possible tumors in under 20 s. This significant difference in processing time and the significant potential of improving the results by increasing the resolution of the training data is highly promising and should be further studied in future research.

## Discussion

This study aimed to use unsupervised learning to automatically segment areas of fundus images that are related to tumors without the need for prior labeling. The results are evaluated by medical experts, and the newly labeled data are then used to train a supervised segmentation model, a more cost-effective approach than using unsupervised labeling alone. This study’s novel semi-supervised method can utilize a larger sample size of fundus images than similar studies that rely on manual labeling by medical experts. Moreover, the proposed model is also computationally efficient, as it is based on a small number of parameters. It should be noted that there are currently no publicly available datasets of fundus images that focus on retinoblastoma.

As previously mentioned, a medical expert typically carries out the process of labeling pixels associated with retinoblastoma tumors in fundus images, and various factors can influence their results. As a result, it is difficult to establish a standardized process.

Several studies have demonstrated the effectiveness of machine learning and image processing in providing detailed tumor segmentation results across different fields. Machine learning is capable of identifying similarities between human judgments, making it a powerful tool for creating data-driven models. While some researchers have focused on presenting simpler approaches, such as manual thresholding for tumor segmentation and using only fully connected layers in a classifier^19^, others have preferred to utilize medical-specific machine learning approaches. For example, 2.75D Convolutional Neural Networks have been shown to demonstrate excellent results in both retinoblastoma detection and tumor segmentation^18^, leading to the development of user-friendly applications and tools like MDEyeCare and CRADLE^20^. It is worth noting that the detection of retinoblastoma, regardless of its type, is a binary task that can be accomplished by various approaches, including less-known methods like extreme learning^21^. On the other hand, some studies have used machine learning to its full potential in order to detect retinoblastoma, with promising results from structures like the Multi-Thresholding-Based Discriminative Neural Classifier^22^. However, regardless of the type of cancer, tumor segmentation is a more complex task. Therefore, studies focused on retinoblastoma tumor segmentation have developed more complex algorithms. Some studies have chosen to focus on retinoblastoma detection, with tumor segmentation considered a secondary result of the suggested method^23^. While these studies have provided practical models in this case, other studies have prioritized retinoblastoma tumor segmentation as their main goal. For instance, the combination of methods like multi-view convolutional neural networks with MRI data has provided excellent results^24^.

The current proposed method achieved an average Sørensen–Dice coefficient of 0.93 and an accuracy of 0.89. While the suggested approaches have their limitations, the results of this study suggest that segmenting tumors using semi-supervised learning is a viable option. Additionally, with access to more powerful hardware capable of processing higher-resolution images, it may be possible to detect even smaller signs of retinoblastoma. Although segmenting tumors in fundus images alone is only beneficial for tracking the changes following treatment, combining the network with a classifier could potentially improve the accuracy of retinoblastoma detection. Some studies have focused on recognizing retinoblastoma or its type and have presented heatmaps indicating important parts of the input images. However, their models demonstrated vague boundaries around the tumor. By providing more accurate boundaries, it may be possible to enhance the performance of recognition models.

There are limitations to our study: as mentioned previously, the methods presented do not directly yield recognition results regarding all aspects of the retinoblastoma detection. Specifically, these models do not offer specific information regarding distinctions between treated and untreated tumors, as well as various types of regression or grouping of the affected eye. However, employing the introduced approaches can potentially eliminate extraneous details from fundus images, thereby enhancing the accuracy of subsequent steps needed in retinoblastoma detection including the identification of its type, stage, and treatment-related specifics for future studies.

## Conclusion

This study utilized over 4000 unlabeled fundus images to label pixels in an unsupervised process. The labeled data was then used to train a CNN-based model capable of providing similar results in a more cost-effective manner. Although the unsupervised method from the first approach drew more precise tumor boundaries the secondary model, trained using original images and results of unsupervised method is preferred due to its lower cost and higher probability of ignoring the disc in the fundus image. The second model provides two different outputs, each of which is useful in different situations where processing time is limited or a medical expert is unavailable. To improve the accuracy of tumor detection, it is necessary to use more complex models based on higher-resolution images and more capable hardware. Although this study does not yield perfect results regarding retinoblastoma tumor segmentation, the proposed models can aid in segmenting the cancerous lesions more efficiently and effectively.

## Data Availability

The datasets generated and analyzed during the current study are not publicly available due to the Iran University of Medical Sciences’ protocol but are available from the corresponding author upon reasonable request.
